# Acute Myeloid Leukemia Epigenetic Immune Escape From Nature Killer Cells by ICAM-1

**DOI:** 10.3389/fonc.2021.751834

**Published:** 2021-10-13

**Authors:** Yang Xiao, Jinghong Chen, Jia Wang, Wei Guan, Mengzhen Wang, Linlin Zhang, Zhiding Wang, Lixin Wang, Li Yu

**Affiliations:** ^1^ Department of Hematology and BMT Center, Chinese PLA General Hospital, Beijing, China; ^2^ Department of Hematology and Oncology, International Cancer Center, Shenzhen Key Laboratory, Shenzhen University General Hospital, Shenzhen University Health Science Center, Shenzhen University, Shenzhen, China; ^3^ Beijing Institute of Basic Medical Sciences, Beijing, China

**Keywords:** AML, ICAM-1, NK, methylation, immune escape

## Abstract

Acute myeloid leukemia (AML), a malignant disorder of hemopoietic stem cells. AML can escape immunosurveillance of natural killer (NK) by gene mutation, fusions, and epigenetic modification, while the mechanism is not clearly understood. Here we show that the expression of Intercellular adhesion molecule‐1 (ICAM‐1, CD54) is silenced in AML cells. Decitabine could upregulate ICAM-1 expression, which contributes to the NK-AML cell conjugates and helps NK cells kill AML cells. We also show that ICAM-1 high expression can reverse the AML immune evasion and activate NK cells function *in vivo*. This study suggests that a combination of the hypomethylating agent and NK cell infusion could be a new strategy to cure AML.

## Highlights

- AML can escape immunosurveillance. The mechanism of AML immune evasion is not clearly understood.
- The expression of ICAM-1 is silenced, which could be reversed by decitabine. Thus, decitabine can help NK cells recognize and kill AML cells, which reverses AML immune evasion.
- This study suggests that the hypomethylating agent decitabine in combination with NK cell infusion may be a working strategy to cure AML.

## Background

AML is a heterogeneous disease from the biological and clinical standpoint with increasing incidence, high mortality, and a very poor prognosis (1, 2). Current therapies show also a high rate of relapse (3). AML has neoplastic changes and clonal proliferation due to gene mutation, fusions, and epigenetic modification, ultimately resulting in the inhibition of normal hematopoiesis and escape from immunosurveillance (4).

Immunotherapy based on mechanisms of immune surveillance has been recognized as a potential therapeutic strategy for numerous cancer elimination (5). Immunotherapy with strategies aimed at boosting the immune response has pushed NKs into the spotlight (2). Intercellular adhesion molecule‐1 is a membrane glycoprotein of the Ig superfamily and plays an important role in inflammatory processes and immune responses (6). Studies showed that promoting the NK-AML cell conjugate formation by upregulating lymphocyte-function associated (LFA) antigen expression on NK cells and by inducing ICAM-1 expression on AML cells could increase their cytotoxic activities (7). Thus, restoring ICAM-1 expression in AML may combine the benefit of targeting AML cells and NK-mediated killing. However, limited studies are relating to how to increase the ICAM-1 expression on the surface of AML cells.

Decitabine is a valuable treatment option in AML patients (8). An important mechanism of tumor immune response evasion by cancer cells lies in their ability to display the loss of antigenicity, resulting in less potent for immune cells and substances in cancer elimination (5). A hypomethylating agent has favorable effects on anti-tumor immune response by reactivating the tumor suppressor genes (9). Hypomethylating agents such as decitabine have favorable effects on anti-tumor immune evasion response and limit the ability of cancer cells to alter the expression of tumor-associated antigens by regulating a range of immunomodulatory pathway-related genes (10).

In our previous studies, we found AML was epigenetic silenced (11–13) and could escape immunosurveillance by CD80 (14) and CD48 (15, 16). In this study, we found ICAM-I was also epigenetic silenced in AML and escaped the NK cell killing function. Decitabine is implicated in the regulation of ICAM-1 expression and reverses the AML-NK dysfunction.

## Materials and Methods

### Mice

Male BALB/c mice (6- to 8-weeks-old) were obtained from SPF (Beijing) Biotechnology. All the mice were bred and maintained in the Laboratory Animal Center of Chinese PLA General Hospital, under specific pathogen-free conditions and were treated in strict compliance with the guidelines for the care and use of laboratory animals set out by the Laboratory Animal Center of Chinese PLA General Hospital, the protocol was approved by the Committee on the Ethics of Animal Experiments of Chinese PLA General Hospital (16). All the efforts were made to minimize suffering.

For the *in vivo* study, mice were separated into three groups (five each), and each mouse was injected intravenously (i.v.) with WEHI-3 (2.5×10^5^) on Day 1. For decitabine treatment, each mouse was injected intraperitoneally (i.p.) 0.5 mg/kg/day from Day 1 to Day 3. For NK cell infusion, NK cells were separated from the spleen by the NK Cell Isolation Kit (Miltenyi), and each mouse was injected intravenously (i.v.) with 1×10^6^ NK cells on Day 3. For the observation of the tumor burden in the mouse spleen and liver, the mice were sacrificed by CO_2_ inhalation on Day 17 after WEHI-3 injection, when the mice of the control group started to paralyze and be dying. Isoflurane inhalation was used for any anaesthetization. Spleen and liver were stained with H&E for histological analysis.

### Database Analysis

173 AML patients’ and 70 healthy donors’ samples were analyzed the ICAM-1 RNA sequencing expression data of The Cancer Genome Atlas (TCGA) database by GEPIA (17).

### Cell Culture

Human cell lines NK92, HL60, and NB4 cells were maintained in RPMI 1640 medium and mouse cell line WEHI-3 cells were maintained in DMEM medium, supplemented with 50 μg/mL streptomycin, 50 IU penicillin, and 10% fetal bovine serum. All the cell lines are obtained from ATCC and culture at 37°C in 5% CO_2_. For decitabine stimulation, the cell lines were cultured with decitabine for 72 h and decitabine was re-supplement every 24 h. Human cell lines (HL60 and NB4) were cultured with 1 μmol/mL decitabine. The mouse cell line WEHI-3 was cultured with 0.25 μmol/mL decitabine.

### RNA Extraction and Analysis

Total RNA was extracted from cells using the TRIzol RNA Isolation Reagents (Thermo Fisher Scientific). RNA was reverse-transcribed in a 25 μL reaction volume using AMV Reverse Transcriptase (Promega), and then cDNA was amplified using KAPA SYBR FAST qPCR Kits (Kapa Biosystems). The relative expression of the gene of interest was determined using the 2–ΔΔCt method, with GAPDH as the internal control (18). The primers used were: Human ICAM-1: Forward: GGCATTGTTCTCTAATGTCTCCG, Reverse: GTCGAGCTTTGGGATGGTAG; Mouse ICAM-1: Forward: TTGGGCATAGAGACCCCGTT, Reverse: GCACATTGCTCAGTTCATACACC; 18S: Forward: TTGACGGAAGGGCACCACCAG, Reverse: CATACCAGGAAATGAGCTTGA.

### Cytometric Analysis

All the cell experiments were prepared on ice and cells were washed with FACS buffer. All the samples were incubated with 2.4G2 anti-Fc receptors (BD Pharmingen) before incubation with other antibodies. Fluorescence conjugated anti-mouse CD54 (Biolegend, YN1.7.4) and Pacific Blue anti-human ICAM-1 (Biolegend, HA58) antibodies were used. All the flow cytometry data were acquired with NAVIOS (BECKMAN) and analyzed by FlowJo software (Tree Star).

### NK Cells Isolation

The NK Cell Isolation Kit (Miltenyi) was used for the untouched isolation mouse NK cells from spleen cells, which were activated by IL-2 and incubated in 5% CO_2_ for 24 hours.

### The Adhesion Between NK Cells and WEHI-3 Cells

NK cells were divided into PBS and Decitabine (DAC) groups. The WEHI-3 cells that were treated or untreated with decitabine from Day 1 to Day 3 were collected on Day 4. The PBS and DAC groups of WEHI-3 were added into each well of NK cells. WEHI-3 untreated with decitabine and without NK cells was considered as a blank group for cytometric analysis.

### NK Killing Assay

NK killing assay was described previously (16). In brief, the control cells were stained with CellTrace CFSE Cell Proliferation Kit (ThermoFisher Scientific), and the Decitabine treated cells were stained with CellTrace Far-red (ThermoFisher Scientific). Then the control cells and the Decitabine treated cells were plated on a 96-well plate. For ICAM-1 blocking, 20 mg/mL of anti-mouse ICAM-1 antibody (Biolegend, YN1/1.7.4) were added to each well. Then NK cells were added to each well and 20-24 h later the samples were analyzed by Flow Cytometer. Human cell lines (HL60 and NB4) were co-cultured with NK92 cells. The mouse cell line WEHI-3 was cultured with NK cells separated from mouse. The No NK group as control, the ratio of NK group was decitabine treated and untreated cell co-culture with NK cells. The CON and anti-ICAM-1 groups were percentages (1- normalized Treated/Untreated). The CON means the specific killing of decitabine treated and untreated cell co-culture with NK cells. The anti-ICAM-1 group means the specific killing of decitabine treated and untreated cell co-culture with NK cells and ICAM-1 antibody blocking.

### Statistical Analysis

Data were expressed as the mean ± standard deviation. Differences between groups were analyzed using the t-test. The mouse model survival analysis was showed as Kaplan-Meier. A P-value less than 0.05 was considered to be significant. GraphPad Prism software (version 7.00) was used for All the statistical procedures. All the flow cytometry data were analyzed by FlowJo-V10 software.

## Results

### ICAM-1 Silenced in AML Patients and Reversed by Hypomethylating

**Figure 1:**
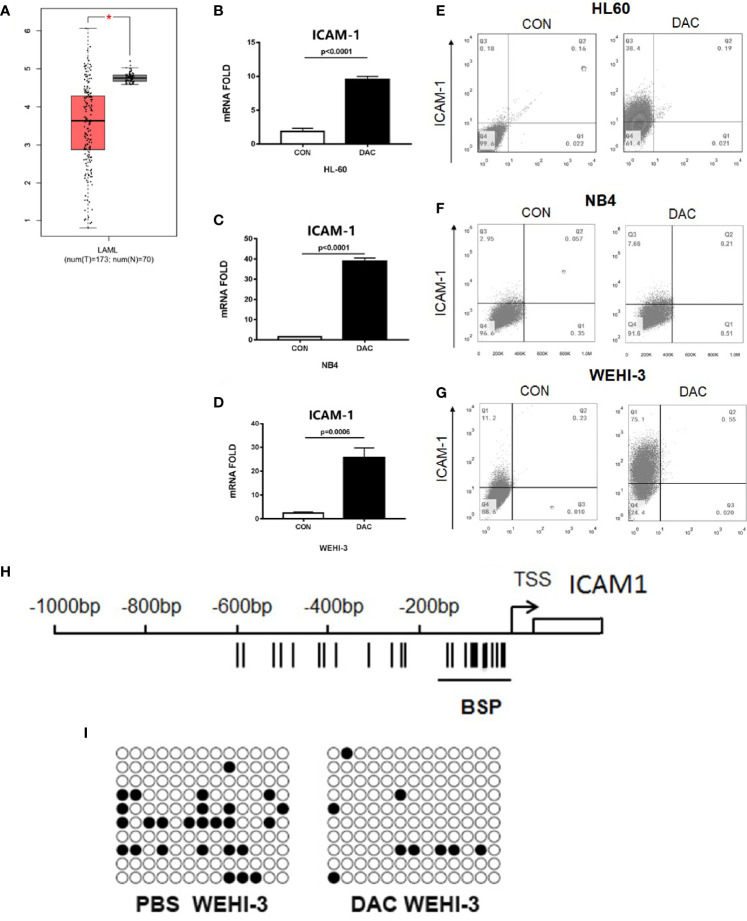
ICAM-1 silenced in AML patients and reversed by hypomethylating. **(A)** The ICAM-1 mRNA Chip analysis of 70 healthy controls (N) and 173 AML patients (T) (TCGA). Q-PCR anlysis of ICAM-1 mRNA expression in **(B)** HL-6, **(C)** NB4, and **(D)** WEHI-3 cells treated with or without decitabine (mean ± SD, n=4). FACS analysis of ICAM-1 expression in **(E)** HL60, **(F)** NB4, and **(G)** WEHI-3 (n=3, typical data). **(H)** Bisulfite sequencing PCR of mouse ICAM-1 methylation sequencing fragment. **(I)** Methylation rate of WEHI-3 treated and untreated with DAC reached 20% [left] and 6.92% [right], respectively.

To determine the role of ICAM-1 in the AML patients, ICAM-1 mRNA expression was analyzed in the bone marrow or peripheral blood of 70 healthy controls and 173 AML patients from The Cancer Genome Atlas (TCGA) by GEPIA (16, 17). ICAM-1 mRNA expression was significantly lower in patients than in normal healthy individuals (**Figure 1A**). ICAM-1 expression on AML cells could increase NK cells cytotoxic activities. reversed the ICAM-1 expression could inhibit the AML immune escape. Q-PCR analysis showed that the hypomethylating agent decitabine can increase ICAM-1 mRNA expression on AML cell lines HL60 (p < 0.0001), NB4 (p < 0.0001), and WEHI-3 (p = 0.0006). The Q-PCR results (**Figures 1B**–**D**) showed that decitabine increased ICAM-1 mRNA expression, which was confirmed by FACS analysis for protein expression on the surface of HL60, NB4, and WEHI-3 cells (**Figures 1E**–**G**). These findings indicate that decitabine increases ICAM-1 expression and reveals a novel mechanism of a therapeutic hypomethylating agent for AML. By the Bisulfite Sequencing PCR analysis, WEHI-3 gene promoter methylation was decreased by decitabine treatment (**Figures 1H, I**). Thus, this hypomethylating agent could increase the ICAM-1 expression by decrease promoter methylation. Thus, decitabine may restore ICAM-1 expression and inhibit AML immune evasion from NK cells.

### Decitabine Inhibits AML Immune Escape From NK Cells by ICAM-1

**Figure 2:**
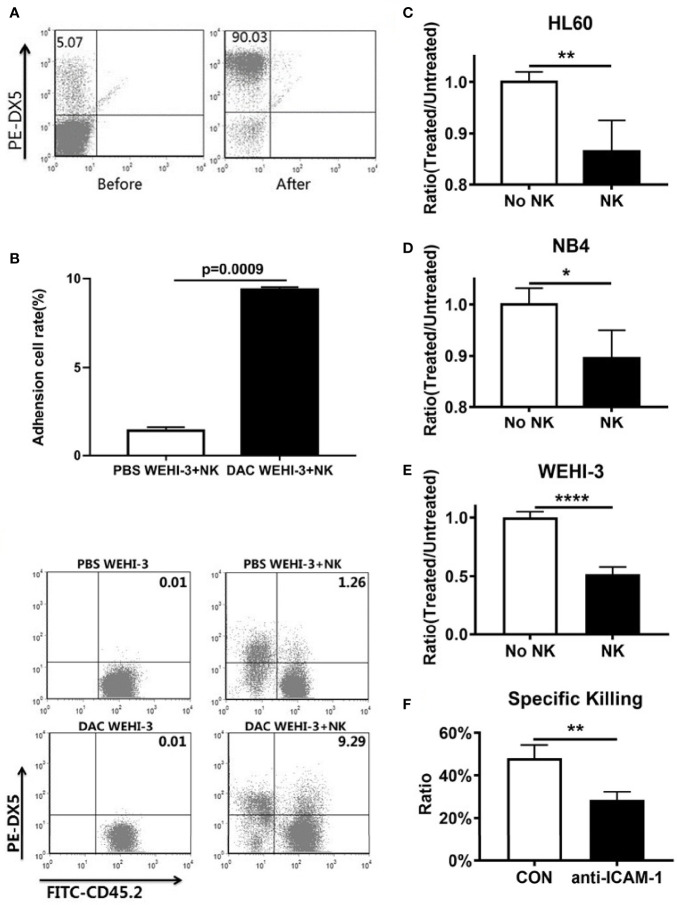
ICAM-1 expression and NK killing rate increased by hypomethylating *in vitro*. **(A)** Purity of NK cells before and after magnetic bead sorting. **(B)** The adhesion between NK cells and DAC WEHI-3 cells was significantly enhanced (mean ± SD, n=3, repeat three times). DAC enhanced NK cells’ sensitivity to **(C)** NB4, **(D)** HL60, and **(E)** WEHI-3, cells (mean ± SD, n=4). **(F)** The NK cell killing rate of decitabine treated or untreated with WEHI-3 cells, and then co-cultured with NK cells with or without the ICAM-1 antibody to block ICAM-1 for 24h (mean ± SD, n=4). *p < 0.05, **p < 0.01, ****p < 0.0001.

To determine if decitabine influence the NK cell to find AML, WEHI-3 cells were treated or untreated with decitabine and co-cultured with sorted NK cells (**Figure 2A**). FACS analysis showed that the adhesion ratio between NK cells and WEHI-3 treated with DAC was enhanced (p = 0.0009), with the PBS as the control group (**Figure 2B**). The cells that adhere to NK cells account for 9.29% among the WEHI-3 cells treated with DAC, while 1.26% among the WEHl-3 cells treated with PBS. And the data showed that the NK cell killing rate increased significantly. NK cells can kill more HL60 (**Figure 2C**), NB4 (**Figure 2D**) and WEHI-3 (**Figure 2E**) cells treated with decitabine than cells treated with PBS. To determine whether decitabine inhibits AML immune escape through ICAM-1 *in vitro*, WEHI-3 cells were treated with or without decitabine, then co-cultured with mouse NK cells with or without the ICAM-1 antibody to block ICAM-1. The NK cell killing function was inhibited by ICAM-1 antibody blockage (**Figure 2F**). Thus, decitabine could increase NK cell killing *via* ICAM-1 *in vitro*.

### AML Immune Evasion Was Decreased by ICAM-1 *In Vivo*

**Figure 3:**
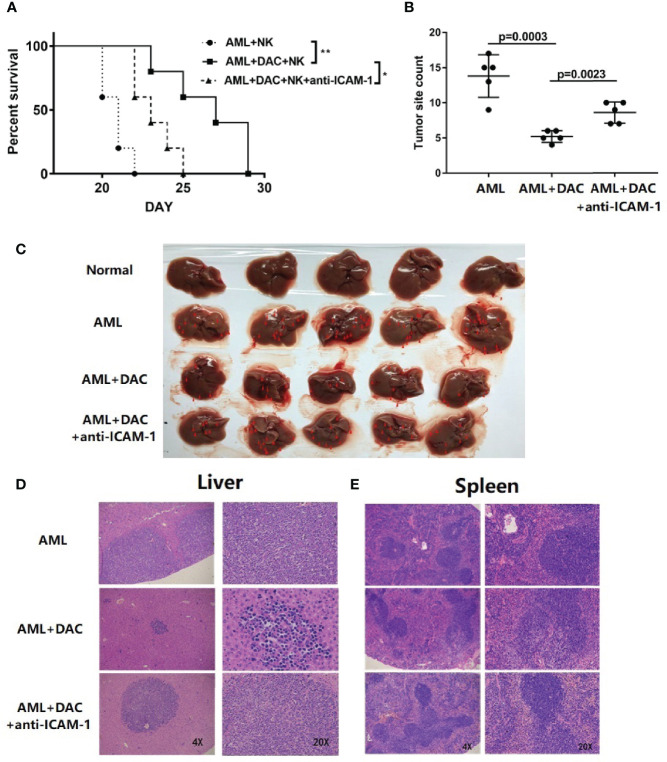
ICAM-1 inhibits AML immune escape *in vivo*. **(A)** Survival of BALB/c mice injected with WEHI-3 cells supplemented or not supplemented with ICAM-1 antibody on Day 1, and then injected decitabine from Day 1 to Day 3, finally injected with NK cells on Day 3 (n=5). **(B, C)** The tumor site and count of mouse livers on 17 days after injection (mean ± SD, n=5). H&E staining of **(D)** livers and **(E)** spleen. *p < 0.05; **p < 0.01.

To determine whether decitabine could inhibit AML immune escape *in vivo*, BALB/c mice were injected with WEHI-3 and mouse NK cells, and their survival and tumor burden was monitored. The mice were randomly divided into control, DAC, and DAC + ICAM-1 antibody groups (n = 5). Firstly, to assessing survival, the mice were bred until the first signs of paralysis determined the end of observation for each mouse. The survival of the DAC group was significantly longer compared to the control (**Figure 3A**, Kaplan-Meier, p = 0.0019). When injected with the ICAM-1 antibody, the survival time of the DAC + ICAM-1 antibody group decreased compared to the DAC group (p = 0.0278). These findings indicate that decitabine could increase mouse survival time *via* ICAM-1 *in vivo*. To assess tumor burden and invasion in the spleen, the mice were sacrificed on the 17th day after the WEHI-3 injection. After WEHI-3 inoculation, the AML cells invaded the spleen and liver, formed extramedullary masses. The number of tumor masses in the DAC group decreased compared to the control group (p = 0.0003) (**Figures 3B, C**). When injected with the ICAM-1 antibody, the number of tumor masses of the DAC group was less than the DAC + ICAM-1 antibody group (p = 0.0023) (**Figures 3B, C**). H&E staining of livers (**Figure 3D**) and spleens (**Figure 3E**) showed more tumor masses and invasion than the DAC group. Thus, decitabine could inhibit AML immune evasion in the spleen *in vivo* and improve mouse survival by increasing ICAM-1 expression on the AML cell surface and enhancing NK cell killing function.

## Discussion

This is the first study that implicates methylation in the regulation of AML ICAM-1 expression, and we show that the hypomethylating agent decitabine could increase ICAM-1 expression, which in turn reverses AML immune evasion from NK cells.

Epigenetic modification in cancers is critical for the immune cell interactions, which including DNA, histone, and chromatin structure modifications (19). Emerging evidence and our works (15, 16) show that tumors could use various epigenetic mechanisms to immune escape. Epigenetic targeting agents are becoming attractive immunomodulatory drugs and will have major impacts on immunotherapy. Tumor epigenetics down-regulation antigen-presenting (20) and other immune molecular, which become invisible to T cell and other immune cells. Hypermethylation can reverse the MHC-I antigen presentation (21). In our previous studies, AML cells can escape immunosurveillance of NK cells by downregulating CD48 expression on AML cell surface (16). AML cells with downregulated CD48 through epigenetic modification increase DNA methylation and decrease histone acetylation (15).

NK cells play a vital role in AML eradication. Increased ICAM-1 expression contributes to the NK-AML cell conjugates and helps NK cells kill AML cells. Other mechanistic studies also reveal that the increased cytotoxic activity correlates with an increased conjugate formation by upregulating LFA expression on NK cells and by inducing ICAM-1 expression on AML cells (7).

The expression of ICAM-1 on AML cells is silenced, while our findings showed that decitabine could upregulate ICAM-1 expression on AML cells and inhibit AML immune evasion. The mechanism of hypomethylating agent decitabine on ICAM-1 expression is still unclear and required to be explored. Our findings indicated that decitabine may be potentially utilized to modulates the immune system and help to cure AML with other drugs.

## Data Availability Statement

The original contributions presented in the study are included in the article/supplementary material. Further inquiries can be directed to the corresponding authors.

## Ethics Statement

The animal study was reviewed and approved by the Committee on the Ethics of Animal Experiments of Chinese PLA General Hospital.

## Author Contributions

Conception and design of the study: LY, LW, and ZW. Acquisition, analysis, and interpretation of data: YX, JC, and JW. Contribution of administrative, experimental, analytic, or material support: WG, MW, and LZ. Writing-Original Draft Preparation: YX and JC. Editing: ZW. All authors contributed to the article and approved the submitted version.

## Funding

Chinese National Major Project for New Drug Innovation (2019ZX09201002003). National Natural Science Foundation of China (82030076, 2070161, 81970151, 81670162 and 81870134). Shenzhen Science and Technology Foundation (JCYJ20190808163601776, JCYJ20200109113810154). Shenzhen Key Laboratory Foundation (ZDSYS20200811143757022). Sanming Project of Shenzhen (SZSM202111004).

## Conflict of Interest

The authors declare that the research was conducted in the absence of any commercial or financial relationships that could be construed as a potential conflict of interest.

## Publisher’s Note

All claims expressed in this article are solely those of the authors and do not necessarily represent those of their affiliated organizations, or those of the publisher, the editors and the reviewers. Any product that may be evaluated in this article, or claim that may be made by its manufacturer, is not guaranteed or endorsed by the publisher.
